# Local Conformational Changes in the DNA Interfaces of Proteins

**DOI:** 10.1371/journal.pone.0056080

**Published:** 2013-02-13

**Authors:** Tomoko Sunami, Hidetoshi Kono

**Affiliations:** Molecular Modeling and Simulation Group, Quantum Beam Science Directorate, Japan Atomic Energy Agency, Kizugawa, Kyoto, Japan; University of South Florida College of Medicine, United States of America

## Abstract

When a protein binds to DNA, a conformational change is often induced so that the protein will fit into the DNA structure. Therefore, quantitative analyses were conducted to understand the conformational changes in proteins. The results showed that conformational changes in DNA interfaces are more frequent than in non-interfaces, and DNA interfaces have more conformational variations in the DNA-free form. As expected, the former indicates that interaction with DNA has some influence on protein structure. The latter suggests that the intrinsic conformational flexibility of DNA interfaces is important for adjusting their conformation for DNA. The amino acid propensities of the conformationally changed regions in DNA interfaces indicate that hydrophilic residues are preferred over the amino acids that appear in the conformationally unchanged regions. This trend is true for disordered regions, suggesting again that intrinsic flexibility is of importance not only for DNA binding but also for interactions with other molecules. These results demonstrate that fragments destined to be DNA interfaces have an intrinsic flexibility and are composed of amino acids with the capability of binding to DNA. This information suggests that the prediction of DNA binding sites may be improved by the integration of amino acid preference for DNA and one for disordered regions.

## Introduction

Protein–DNA interaction plays an essential role in many cellular functions such as transcription, replication, recombination, and DNA packaging. To understand the recognition mechanisms of individual DNA binding proteins, the protein structures of DNA-bound as well as DNA-free forms have been analyzed [1], [2], [3], [4], [5]. It has been reported that flexible regions undergo conformational changes in order to recognize specific DNA targets [4], [5], [6], [7], [8], [9], [10]. For example, the β2/β3 connecting loop of the papillomavirus E2 protein, which is unstructured in the free form, adopts a β-hairpin conformation in order to form electrostatic contacts with DNA backbone phosphates in the complex form [4], [6]. The conformational change in the loop has also been observed in molecular dynamics simulations [7], [8]. Another example of conformational change was observed in the linker region of MATα2 [5]. In this case, two independent copies of the complex were found in the asymmetric unit. The flexible linker [9] in one copy of MATα2 adopted an α-helix structure and the other adopted a β-strand structure. The sequence of the region is coined as a chameleon sequence. This conformational transition at the sequence is thought to be important for DNA recognition [10].

The sequence characteristics of such proteins have also been examined. Dunker and other groups developed methods to predict the intrinsically disordered region of proteins on the basis of X-ray, nuclear magnetic resonance, and circular dichroism spectroscopic data [11], [12]. Such regions are thought to undergo a disordered–ordered transition of the conformations when they interact with a binding partner [11]. The genome-wide application of these methods indicated that transcription factors, especially those in eukaryotes, have a higher amount of intrinsically disordered regions [13], [14]. The proposed role of the regions is to facilitate DNA searching and modulate the specificity and affinity to DNA [15]. Although the conformational change in the flexible region in DNA binding proteins has been well recognized, comprehensive analyses of the local structural rearrangements of proteins upon DNA binding have not been conducted yet.

To assess the structural rearrangement, the classification of the 3D geometries of local protein structures is necessary. Historically, Pauling first proposed the idea that protein structures could be represented as strings of secondary structures [16], and since then, secondary structures have often been used to compare protein structures [17], [18]. However, secondary structures are too coarse to detect subtle local conformations because they only focus on the arrangement of the hydrogen bonding partners of the backbone atoms. The use of structural alphabets was then proposed to more precisely describe local structures, where the alphabets are assigned to certain local conformations [19]. Structural alphabets have been reported to classify protein structures more precisely than secondary structures [20], [21] and have been applied to structure prediction [22], [23], 3D structure comparison [24], [25], motif searches [26], protein–protein interaction analysis [27], [28], and *de Novo* protein design [29].

In this study, quantitative analysis of conformational changes in DNA binding proteins using structural alphabets [30] was performed. Using sets of proteins whose structures were solved for both the DNA-free and DNA-bound forms, it was found that DNA interfaces have higher conformational flexibility than non-interfaces. It was also found that conformationally changed regions in DNA interfaces have a high amount of glycine, proline, and the hydrophilic residues that have previously been found in intrinsically disordered regions [11], [12], [31]. This result indicates that fragments of DNA interfaces are composed of amino acids that have high flexibility and DNA binding capability.

## Materials and Methods

### Data Preparation for DNA-bound and DNA-free Forms

**Figure 1:**
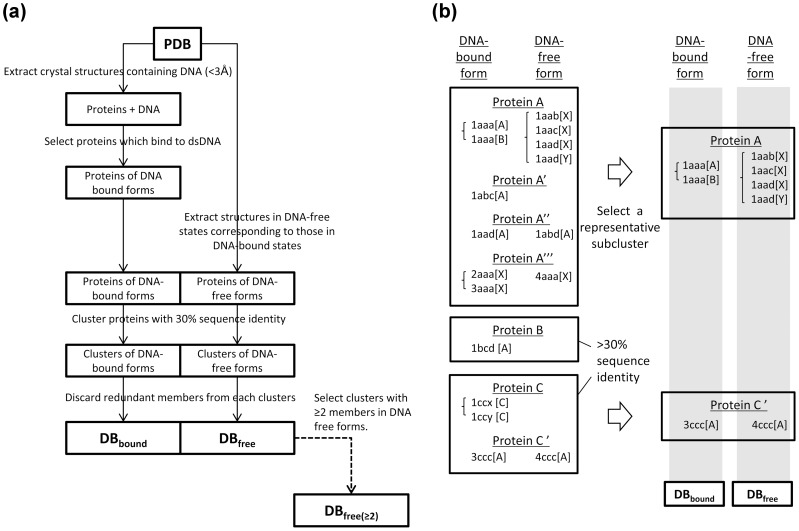
Data set preparation. (a) Workflow for obtaining DB_bound_, DB_free_, and DB_free>2_. (b) Schematic diagram for the preparation of a set of non-redundant clusters.

Non-redundant pairs of crystal structures of DNA-free and DNA-bound forms were prepared as follows. A flow chart of the data preparation and a schematic diagram of the reduction of the dataset redundancy are given in Figure 1.

To prepare the dataset for the DNA-bound forms, the Protein Data Bank (PDB) (December 2010 version [32]) was searched for all DNA complexes with better than 3 Å resolution. The proteins that were co-crystallized with ss-DNA, Z-DNA, and RNA were then discarded. Antibodies, artificial DNA binding proteins, and a structure of a trp repressor that was crystallized with a high concentration of isopropanol (pdbID: 1mi7) were further excluded. The PDB was then searched for the DNA-free forms of the DNA-bound proteins (>90% sequence identity) with a resolution better than 3 Å. To reduce the dataset redundancy, the selected proteins with a sequence identity of 30% were clustered using Blastclust [33]. Subclusters with a sequence identity of 90% were then made within each of the clusters. In each cluster, the representative subcluster was determined to be the one that contained the largest number of protein chains among all subclusters. Finally, 126 representative cluster pairs of DNA-bound forms and DNA-free forms were obtained. Hereafter, these representatives are referred to as DB_bound_ and DB_free_, respectively.

To evaluate the conformational variation in the DNA free forms of the proteins, another dataset was prepared. Clusters that had more than two protein chains were extracted from DB_free_. This dataset is referred to as DB_free(≥2)_ and contained 86 clusters. The members of DB_bound_, DB_free_, and DB_free(≥2)_ are listed in Table S1.

### Assignment of 11 Structural Alphabets

**Figure 2:**
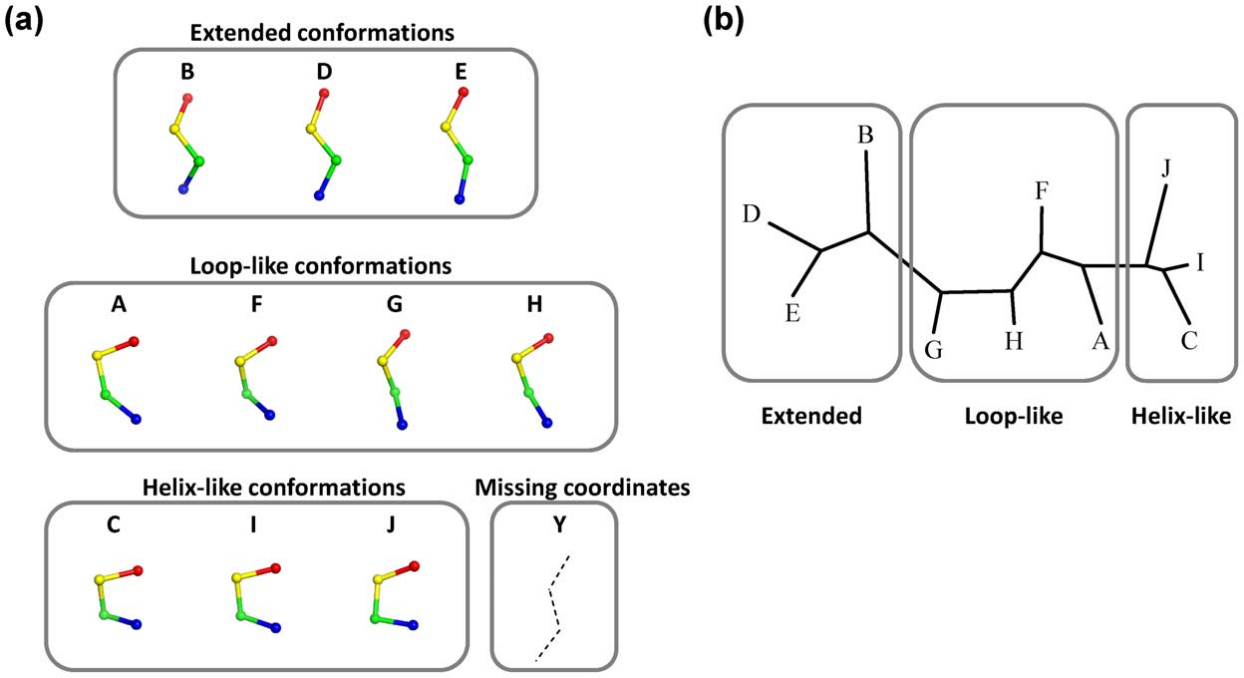
Structure of the eleven fragments. (a) Ten fragments (assigned to A to J) of the 4-residue-long structural alphabet library and a fragment with missing coordinates (assigned to Y). (b) Similarity of the ten alphabet structures calculated using cRMS as the distance with the Fitch in Phylip package. Fragment structures were drawn with Pymol [42].

A library composed of 10 4-residue-long fragments that were developed by Kolodony *et al.* [30] and one fragment we introduced in this study was used to describe the protein structures. For describing the framents, alphabets A to J were assigned (Fig. 2(a)) and their conformations are shown in Table S2. Hereafter, we call them structural alphabets. In addition to the original 10 fragments, one “Y” code was introduced to describe a fragment for which any of the Cα atoms were not determined in the crystal structure. Such fragments are thought to acquire multi-conformations in a crystal. To each structural fragment, the best-matched alphabet in terms of the root mean square deviation of the Cα atoms (cRMS) was assigned. The fragments corresponding to 5% outliers in the cRMS distributions were discarded from the analysis. In addition, fragments were excluded from the analysis if the sequences of the corresponding fragments were not identical among the proteins of the same cluster. To intuitively catch conformational feature of fragments, the ten alphabet structures were further classified into three conformations, extended, loop-like and helix-like conformations based on the cRMS (Fig. 2(b)).

### DNA Interface/non-interface Assignment

A DNA interface residue was one that was exposed to both the solvent as well as the DNA. To determine such residues, the solvent accessible surface area (ASA) of the proteins in the DNA-bound form was first calculated after removing the bound DNA using the ASC program [34]. The relative ASA for each residue was then calculated as the ratio of the surface area of a residue in the protein structure to that of a residue in a Gly-X-Gly tri-peptide having the trans form. The surface residues were defined as those with a relative ASA of more than 20%. The ASA on the protein structure in the presence of the bound DNA was also calculated. If the ASA of the residue exposed to the solvent was different between the DNA-bound and unbound structures, the residue was considered to be within the DNA interface. In addition, if at least one residue of a fragment was judged to be within the DNA interface, the fragment was considered a DNA interface and the remaining fragments were regarded as DNA non-interfaces.

### Conformational Changes Upon DNA Binding

Conformational changes were considered to be the differences in the structural alphabets of the fragments in the DNA-free and the corresponding DNA-bound forms. The probability of the conformational change of fragment *l* from alphabet *i* in DNA free-forms (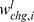) was calculated as follows:
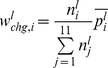
where 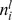 is the number of alphabet *i* assigned to fragment *l* in DNA-free forms. The variable 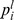 is the probability of fragment *l* having alphabet *i* in the DNA-bound form and is given by



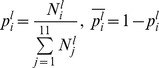
where 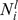 is the number of alphabet *i* assigned to fragment *l* in the DNA-bound forms.

The frequencies of the conformational change in the DNA interfaces (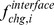) and non-interfaces (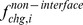) of alphabet *i* were calculated as follows:
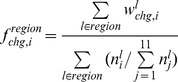
where “region” denotes DNA interface or DNA non-interface hereafter.

The alphabet propensity, or the ratio of the frequencies of the conformational changes in the DNA interfaces and non-interfaces (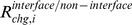) is given by
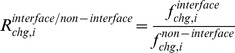



The frequencies of the conformational changes upon DNA binding for any alphabet (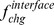 for interfaces and 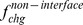 for non-interfaces) were defined as
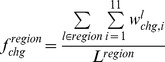
where 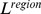 is the number of fragments in region.

### Conformational Variations in the DNA-free Forms

To determine the conformational variations in the DNA-free forms, the intrinsic conformational variation, which can be observed as alphabet variations within the same fragments obtained from different crystal structures, was considered. The expected alphabet variation of fragment *l* (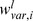) was calculated using the following equation:
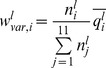
where 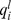 is defined as the probability that two members randomly selected from a set of fragment *l* will both have alphabet *i* and is given as



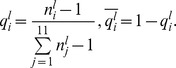



For the disordered conformation “Y,” 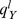 was defined as 1.

The frequencies of the conformational variation in DNA interfaces (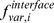) and non-interfaces (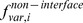) of alphabet *i* were calculated as.
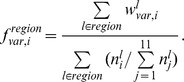



The alphabet propensity of the conformational variation in the DNA interfaces to that in non-interfaces (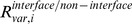) was defined as
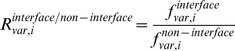



The frequency of the conformational variation for any alphabet (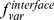 for interfaces and 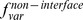 for non-interfaces) was defined as
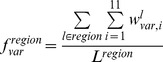
where 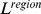 is the number of fragments in region.

### Propensity Calculations

To measure the relative differences in a pair of frequencies, propensities for various pairs of frequencies were calculated. Here, we describe, for example, how residue propensities are calculated. The frequencies of each amino acid *r* in the conformationally changed fragments were calculated for DNA interfaces (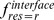) and non-interfaces (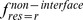) with the following equation:
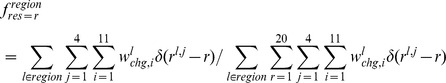

_where_ 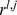 is the *j*th amino acid residue of fragment *l*. The function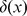 is 1 if *x* is 0 and 0 otherwise.

The frequencies of each amino acid *r* in the conformationally unchanged fragments were calculated for the DNA interfaces (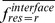) and the surface (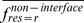) with the following equation:
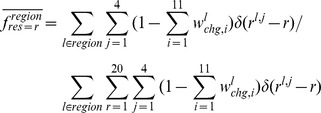



The residue propensities for the DNA interfaces (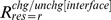) and non-interfaces (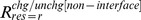) were defined as



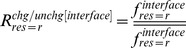
 and 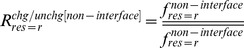



In addition, to measure the importance of each DNA interface residue in the conformationally changed and unchanged fragments, the interface-residue propensities for the conformationally changed fragments (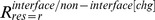) and conformationally unchanged fragments (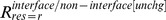) were defined as



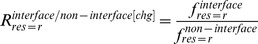
 and 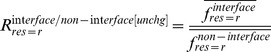



### Alphabet Propensities for the Conformationally Changed Fragments and for Disorder-to-order Conformationally Changed Fragments and Order-to-order Conformationally Changed Fragments

In a similar way as residue propensity, calculated are alphabet propensities in the DNA interfaces (
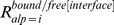
) and non-interfaces (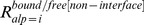) to characterize alphabets induced by conformational changes upon DNA binding, alphabet propensities for the DNA interfaces (
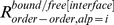
) and non-interfaces (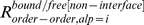) to characterize the order-to-order conformational changes, and alphabet propensities of the fragments that undergo a disorder-to-order conformational change upon DNA binding for the DNA interfaces (
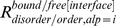
) and non-interfaces (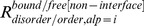). The details for these calculations are provided as supplementary information (Text S1).

### Statistical Reliability

Because the number of protein structures used in this study was limited, the statistical reliability of the calculated values was evaluated. The BCa bootstrap procedure [35] was used to estimate the confidence intervals for frequencies calculations on which propensities were calculated. We constructed 10,000 bootstrap datasets by resampling DB_bound_ and DB_free_. In this test, the reliability standard was set as 85% of a two-sided confidence interval from the average value.

## Results and Discussion

### Dataset Preparation and Structural Alphabet Assignment

One hundred and twenty-six representative pairs of clusters in the DNA-free (DB_free_) and DNA-bound (DB_bound_) forms were obtained with a sequence similarity of less than 30%. The representative clusters were a set of subclusters with the largest members within each cluster (Table 1 and Table S1). The proteins of the 126 clusters had dsDNA binding domains that belonged to different structural classes according to the SCOP classification (version 1.75)[36]: 43 all alpha proteins, 12 all beta proteins, 30 alpha and beta proteins (α/β), 22 alpha and beta proteins (α+β), 11 multi-domain proteins (α andβ), 1 small protein, and 1 coiled coil protein. The remaining 32 proteins were not classified in the SCOP database.

**Table 1:** Database composition.

Cluster ID	Representative PDB ID(chain)* in DB_free_	Representative PDB ID(chain) * in DB_bound_	Molecular name**	Used for DB_free(≥2)_ ***
**1**	1aqi(A)	2ih2(A)	Modification methylase TaqI	✓
**2**	1aro(P)	1cez(A)	Bacteriophage T7 RNA polymerase	
**3**	1az3(A)	1sx5(A)	Type II restriction enzyme EcoRV	✓
**4**	1b24(A)	2vs7(A)	Homing endonuclease I-DmoI	
**5**	1bam(A)	3bam(A)	Restriction endonuclease BamHI	
**6**	1baz(A)	1bdt(A)	Gene-regulating protein Arc	✓
**7**	1bjt(A)	3l4j(A)	DNA topoisomerase 2	✓
**8**	1bm9(A)	1f4k(A)	Replication termination protein	✓
**9**	1bpe(A)	2fmp(A)	DNA polymerase β	
**10**	1ci4(A)	2bzf(A)	Barrier-to-autointegration factor	✓
**11**	1ci6(B)	1h89(A)	CAAT/enhancer binding protein β	
**12**	1cmb(A)	1mjo(A)	Methionine repressor	✓
**13**	1eaq(A)	1h9d(A)	Core-binding factor α subunit1	✓
**14**	1es8(A)	1dfm(A)	Endonuclease BglII	
**15**	1eto(A)	3jrh(A)	DNA-binding protein fis	✓
**16**	1ev7(A)	1iaw(A)	Type II Restriction enzyme NAEI	✓
**17**	1evx(A)	1a73(A)	INTRON 3 (I-Ppo) ENCODED ENDONUCLEASE	✓
**18**	1f9f(A)	1jj4(A)	Regulatory protein E2	✓
**19**	1fc3(A)	1lq1(A)	Stage 0 sporulation protein A	✓
**20**	1fr2(B)	1v15(A)	Colicin E9	✓
**21**	1fvi(A)	2q2t(A)	Chlorella virus DNA ligase	✓
**22**	1fx7(A)	1u8r(A)	Iron-dependent repressor IdeR	✓
**23**	1gxq(A)	1gxp(A)	Phosphate regulon transcriptional regulatory protein	
**24**	1hmy(A)	2c7p(A)	Modification methylase HhaI	✓
**25**	1hw5(A)	1zrf(A)	Catabolite gene activator	✓
**26**	1ih7(A)	3nae(A)	DNA polymerase	✓
**27**	1ii7(A)	3dsd(B)	DNA double-strand break repair protein Mre11	✓
**28**	1ikn(A)	2ram(A)	Transcription factor NF-κB p65	✓
**29**	1jbg(A)	1r8d(A)	Transcription activator MtaN	
**30**	1jeq(A)	1jey(A)	Ku70	
**31**	1jeq(B)	1jey(B)	Ku80	
**32**	1jg7(A)	1m5r(A)	DNA β-glucosyltransferase	✓
**33**	1jhg(A)	1tro(A)	Trp repressor	✓
**34**	1jih(A)	3mfi(A)	DNA polymerase η	✓
**35**	1jjh(A)	2bop(A)	E2	✓
**36**	1jye(A)	1efa(A)	Lac repressor	✓
**37**	1k0z(A)	3pvi(A)	PvuII endonuclease	✓
**38**	1ko9(A)	1m3q(A)	8-Oxoguanine DNA glycosylase	
**39**	1ku3(A)	1rio(H)	Sigma factor SigA	
**40**	1mij(A)	1xpx(A)	Protein prospero	
**41**	1mml(A)	3fsi(A)	MMLV Reverse transcriptase domain	✓
**42**	1mpg(A)	3cw7(A)	DNA-3-methyladenine glycosylase 2	✓
**43**	1mug(A)	1mwi(A)	G/U mismatch-specific DNA glycosylase	
**44**	1okr(A)	1sax(A)	Methicillin resistance regulatory protein mecI	✓
**45**	1ouo(A)	1oup(A)	Vibrio vulnificus nuclease	
**46**	1owl(A)	1tez(A)	Deoxyribodipyrimidine photolyase	✓
**47**	1p7i(A)	2hdd(A)	Engreiled homeodomain	✓
**48**	1q0s(A)	1yf3(A)	DNA adenine methylase	
**49**	1q3b(A)	1k3x(A)	Endonuclease VIII	✓
**50**	1q8i(A)	3k59(A)	DNA polymerase II	✓
**51**	1qht(A)	2vwj(A)	Thermococcus gorgonarius DNA polymerase	✓
**52**	1qtw(A)	2nq9(A)	Endonuclease 4	✓
**53**	1r69(A)	1per(L)	434 repressor	
**54**	1sdo(A)	2p0j(A)	BstYI	
**55**	1tzy(A)	1kx5(C)	Histone H2A.1	✓
**56**	1tzy(B)	1kx5(D)	Histone H2B.2	✓
**57**	1tzy(C)	1kx5(A)	Histone H3	✓
**58**	1tzy(D)	1kx5(B)	Histone H4	✓
**59**	1vhi(A)	1b3t(A)	Nuclear protein EBNA1	✓
**60**	1vok(A)	1qna(A)	Transcription initiation factor TFIID-1	✓
**61**	1vsr(A)	1odg(A)	DNA mismatch endonuclease	
**62**	1w9h(A)	2w42(A)	Archaeal Piwi protein	
**63**	1wtd(A)	1wte(A)	EcoO109IR	✓
**64**	1xhx(A)	2pyj(A)	phi29 DNA polymerase	✓
**65**	1xv5(A)	1y8z(A)	DNA α-glucosyltransferase	
**66**	1xwl(A)	2hhv(A)	DNA Polymerase I	
**67**	1ynm(A)	2fkc(A)	R.HinP1I restriction endonuclease	
**68**	1z91(A)	1z9c(A)	Organic hydroperoxide resistance transcriptional regulator	
**69**	1zbf(A)	3ey1(A)	Ribonuclease H	
**70**	2a40(B)	2dnj(A)	Deoxyribonuclease I	✓
**71**	2a6m(A)	2vih(A)	Transposase ORFA	✓
**72**	2aud(A)	2gig(A)	Type II restriction enzyme HincII	
**73**	2bnk(A)	2c5r(A)	Early protein p16.7	✓
**74**	2ckx(A)	2qhb(A)	Telomere binding protein TBP1	
**75**	2cpg(A)	1b01(A)	Transcriptional repressor CopG	✓
**76**	2d3y(A)	2dp6(A)	Uracil-DNA glycosylase	
**77**	2dt5(A)	3ikt(A)	Redox-sensing transcriptional repressor rex	✓
**78**	2end(A)	2fcc(A)	Endonuclease V	✓
**79**	2f4q(A)	3m4a(A)	Deinococcus radiodurans Type IB DNA topoisomerases	
**80**	2fip(A)	2fio(A)	Late genes activator	✓
**81**	2fok(A)	1fok(A)	FokI restriction endonuclease	✓
**82**	2frh(A)	1fzp(B)	Staphylococcal accessory regulator A	✓
**83**	2fuf(A)	2itl(A)	Large T antigen	✓
**84**	2gpe(A)	2rbf(A)	Bifunctional protein putA	✓
**85**	2gxg(A)	3gfi(A)	ST1710	✓
**86**	2hts(A)	3hts(B)	Kluyveromyces lactis heat shock transcription factor	
**87**	2iru(A)	2r9l(A)	Polymerase Domain from Mycobacterium tuberculosis Ligase D	✓
**88**	2nov(A)	3k9f(A)	DNA topoisomerase 4 subunit A	✓
**89**	2oa9(A)	2oaa(A)	R.MvaI	✓
**90**	2odh(A)	2odi(B)	R.BcnI	
**91**	2ofk(A)	2ofi(A)	3-Methyladenine DNA glycosylase I, constitutive	✓
**92**	2ore(D)	2g1p(A)	DNA adenine methylase	✓
**93**	2p5k(A)	2p5l(C)	Arginine repressor	
**94**	2po4(A)	3c2p(A)	Virion RNA polymerase	
**95**	2qsf(A)	2qsh(A)	DNA repair protein RAD4	
**96**	2rdi(A)	1jx4(A)	DNA polymerase IV (family Y)	✓
**97**	2v1x(A)	2wwy(A)	ATP-dependent DNA helicase Q1	✓
**98**	2ve8(A)	2ve9(A)	DNA translocase FtsK	✓
**99**	2vke(A)	1qpi(A)	Tetracycline repressor	✓
**100**	2wcw(A)	2wiw(B)	Hjc	✓
**101**	2wiu(B)	3dnv(B)	HTH-type transcriptional regulator HipB	✓
**102**	2x6u(A)	2x6v(A)	T-box transcription factor TBX5	
**103**	2yve(A)	2yvh(A)	Transcriptional regulator	✓
**104**	2zd1(A)	3kk1(A)	Reverse transcriptase p66 subunit	✓
**105**	2zkg(A)	3fde(A)	E3 ubiquitin-protein ligase UHRF1	✓
**106**	2znz(A)	2e1c(A)	Putative HTH-type transcriptional regulator PH1519	✓
**107**	3a45(A)	3a46(A)	Formamidopyrimidine-DNA glycosylase	✓
**108**	3bqz(A)	1jt0(A)	Hypothetical transcriptional regulator in QACA 5′region	✓
**109**	3bvq(A)	3c25(A)	NotI restriction endonuclease	✓
**110**	3bvs(A)	3jxy(A)	Alkylpurine DNA glycosylase AlkD	
**111**	3d06(A)	3igk(A)	Cellular tumor antigen p53	✓
**112**	3d1g(A)	3bep(A)	DNA polymerase III subunit β	✓
**113**	3e5u(A)	3e6c(C)	Cyclic nucleotide-binding protein	✓
**114**	3ei3(B)	3ei2(B)	DNA damage-binding protein 2	
**115**	3f0z(A)	3i0w(A)	8-Oxoguanine-DNA-glycosylase	✓
**116**	3fci(A)	1emh(A)	Uracil-DNA glycosylase	✓
**117**	3fhf(A)	3knt(A)	N-glycosylase/DNA lyase	
**118**	3g91(A)	3g00(A)	Exodeoxyribonuclease	✓
**119**	3gn5(A)	3o9x(A)	Uncharacterized HTH-type transcriptional regulator ygiT	✓
**120**	3gva(A)	3gx4(X)	Alkyltransferase-like protein 1	✓
**121**	3gz5(A)	3gz6(A)	MutT/nudix family protein	✓
**122**	3hd0(A)	2w36(A)	Endonuclease V	✓
**123**	3i3q(A)	3o1t(A)	Alpha-ketoglutarate-dependent dioxygenase AlkB	✓
**124**	3iao(A)	1r8e(A)	Multidrug-efflux transporter regulator	
**125**	3lsj(A)	3lsr(A)	DesT	✓
**126**	3mx1(A)	3mx4(A)	Eco29kIR	

* A member which is reported with the highest resolution is shown as a representative. Entire lists of PDB ID (chain ID) of the members were shown in Table S2.

** The molecular names were extracted from the PDB headers of DNA-bound forms. If the molecular names in the headers do not describe the molecule (e.g. Putative protein), the molecular names were extracted from the literatures.

*** ✓ indicates that the clusters were used for DB_free(≥2)_.

The obtained structures were divided into overlapped 4-residue-long fragments and assigned the relevant structural alphabets, and then, the changes in the alphabets in the DNA interfaces and non-interfaces were analyzed. The datasets contained 4963 fragments for the DNA interfaces and 20826 for the non-interfaces. If longer fragments are used, more fragments will be required to express the conformation within the similar range of errors to the 4-residue-long fragments; however, due to the limit of available data, 4-residue-long fragments were used to obtain statistically significant results.

### Fragments in DNA Interfaces Tend to have more Intrinsic Variations in their Conformations than those in DNA Non-interfaces

**Figure 3:**
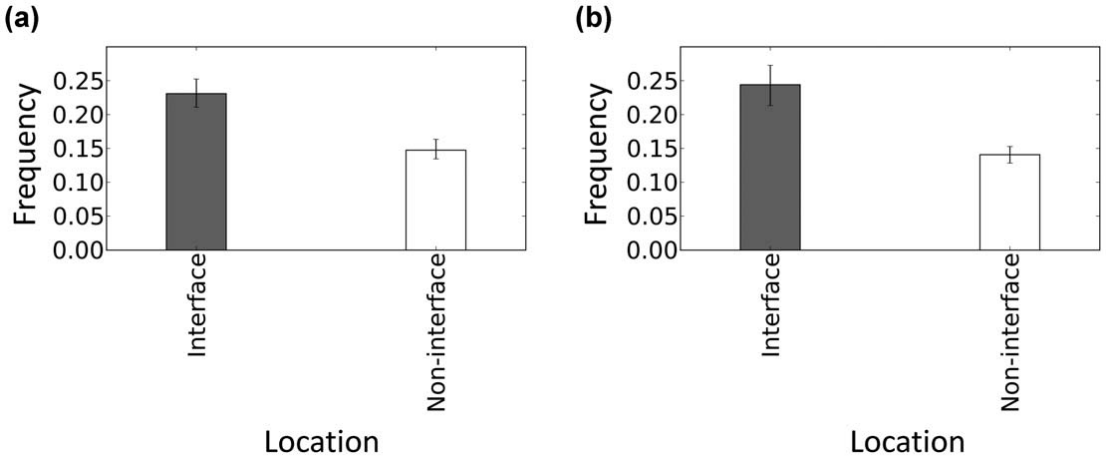
Conformational changes and variations of fragments. (a) Frequencies of conformational change upon DNA binding for DNA interfaces and non-interfaces. (b) Frequencies of conformational variation in the DNA-free forms for DNA interfaces and non-interfaces. Error bar indicates the 85% bootstrap confidence interval.

The frequencies of conformational change upon DNA binding for the DB_free_ and DB_bound_ datasets were calculated. A conformational change was considered to be a change in the alphabet between the DNA-free and DNA-bound forms. The frequencies of conformational changes in the DNA interfaces (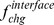) and non-interfaces (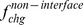) were 23.1% and 14.7%, respectively (Fig. 3 (a)). This result indicates that compared with non-interfaces, DNA interfaces tend to undergo more conformational change upon DNA binding.

It was anticipated that the fragments in DNA interfaces might have a more intrinsic propensity to change conformation in order to adjust to the DNA structure. To examine this assumption, the conformational variations for the fragments in the DNA-free forms were calculated using the dataset DB_free(≥2)_, in which each cluster has at least two members in the DNA-free form. The frequencies of conformational variation in the DNA interfaces (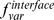) and non-interfaces (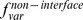) are shown in Fig. 3(b). For the DNA-free forms, the conformational variation in the DNA interfaces (24.4%) was higher than that in the non-interfaces (14.1%), indicating that intrinsic flexibility exists in the DNA interfaces. The flexibility of DNA interfaces was also pointed out in a previous analysis using a small set of DNA binding proteins (7 proteins) [37]. This finding was reconfirmed here using a larger set.

### No Specific Alphabets are Responsible for the Conformational Changes in DNA Interfaces

**Figure 4:**
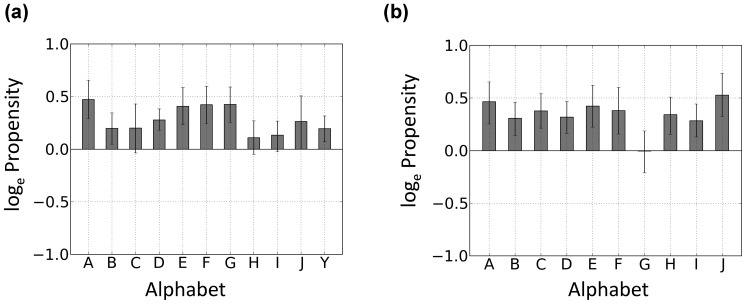
Propensities of the structural alphabets in the DNA interfaces vs. non-interfaces. (a) Conformational change upon DNA binding. (b) Conformational variation in the DNA-free form. Error bar indicates the 85% bootstrap confidence interval.

Next, the differences in the frequency of conformational change for the different alphabets were analyzed to reveal which local structures were affected most often. The alphabet propensities of conformational change in the DNA interfaces to that in the non-interfaces (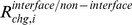;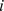 is one of the 11 alphabets) are shown in Fig. 4(a) and those for conformational variation (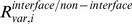) are shown in Fig. 4(b). In Fig. 4(a), a positive value indicates that frequency that alphabet of the free form changes the conformation in DNA interfaces upon DNA binding is higher than that in the non-interfaces. In Fig. 4(b), a positive value indicates that frequency that alphabet has the conformational variation in DNA interfaces is higher than that in non-interfaces. As expected, conformational changes occurred more frequently in the DNA interfaces (positive values in Fig. 4(a)) for all the alphabets, and the alphabets A, D, E, F, G and Y of the 11 alphabets were significantly high. These 6 alphabets are likely to appear more often; however, the errors for B, C, H, I and J are too large to conclude that they significantly appear in the interfaces. The frequencies of the conformational variation in the DNA interfaces were also significantly higher than those for the non-interfaces except for G (Fig. 4(b)) though it is difficult to say which alphabet appears most often owing to the large errors.

### Conformationally Changed Fragments in the DNA Interfaces have Amino Acids Suitable for Producing Flexibility and Binding to DNA

**Figure 5:**
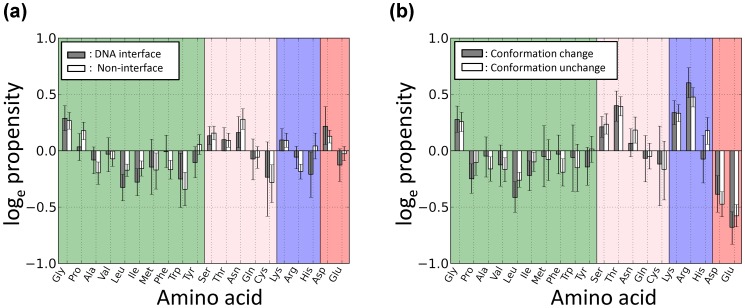
Propensities of the amino acid residues. (a) Conformationally changed fragments vs. conformationally unchanged fragments in DNA interfaces (filled bar) and non-interfaces (open). A positive value indicates that frequency that amino acid is observed in conformationally changed fragments is higher than that in conformationally unchanged fragments. (b) DNA interfaces vs. non-interfaces for conformationally changed fragments (filled) and conformationally unchanged fragments (open). A positive value indicates that frequency that amino acid is observed in DNA interfaces is higher than that in non-interfaces. Error bar indicates the 85% bootstrap confidence interval. The background colors denote the physico-chemical property of amino acids: hydrophobic is shown in pale green; polar in pink; basic in blue; acidic in red.

To reveal whether specific amino acids in the DNA interfaces affect the conformational change upon DNA binding, two pairs of residue propensities were calculated. First, the amino acid propensity in conformationally changed fragments to that in conformationally unchanged fragments (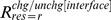
_,_ 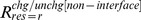) was determined for the DNA interfaces and non-interfaces (Fig. 5(a)). A positive value indicates that frequency that amino acid is observed in conformationally changed fragments is higher than that in unchanged fragments and zero indicates that both frequencies are equal. For example, 1.0 means that frequency of the amino acid in conformationally changed fragments is 2.7 times higher than that in unchanged fragments. Second, the amino acid propensity in the DNA interfaces to that in the non-interfaces was calculated for the conformationally changed and unchanged fragments (Fig. 5(b)).

Figure 5(a) shows that Asn, Gly, Pro, Ser, Asp, Thr and Lys have a positive value, indicating that they are favored amino acids in conformationally changed fragments located in non-interfaces. In contrast, the disfavored amino acids in the conformationally changed fragments of the non-interfaces were Trp, Cys, Ala, Arg, Leu, Met, Phe, Ile, and Val. These results clearly show that hydrophilic residues, Gly, and Pro are located on more flexible fragments in the non-interfaces. This trend was also found in disordered regions [11], [12], [31]. However, there were no significant differences between the propensity in the DNA interfaces and non-interfaces (filled and open bars in the figure), indicating that the conformation change in fragments depends basically on the amino acid types constituting the fragments and not on the positions.

The propensities of the amino acid frequency in the DNA interfaces against that in the non-interfaces for conformationally changed fragments (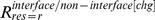) and conformationally unchanged fragments (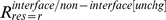) are shown in Fig. 5(b). A positive value indicates that frequency of amino acid in DNA interfaces is higher than that in non-interfaces and zero indicates that both frequencies are equal. The amino acids that favored to interact with DNA in the conformationally unchanged fragments are Arg, Thr, Lys, Gly, Ser, His, and Asn. On the other hand, Glu, Asp, Leu, Phe, Val, Ala, and Ile were disfavored in those fragments. The importance of basic and hydrophilic residues in the DNA interfaces has been emphasized in several previous reports [38], [39], [40]. Gly was also reported to be favored in protein–DNA interfaces but not in protein–protein interfaces [41]. Again, no significant differences could be detected in the propensity of the conformationally changed and unchanged fragments. These findings in amino acid propensities indicate that the amino acid preference depends solely upon the location of a fragment, that is, upon whether it is in a DNA interface or not, and whether a conformational change occurs upon DNA binding depends on the type of amino acids that constitute a fragment.

### Three Specific Alphabets Appear more Often in Conformationally Changed Fragments Located in DNA Interfaces

**Figure 6:**
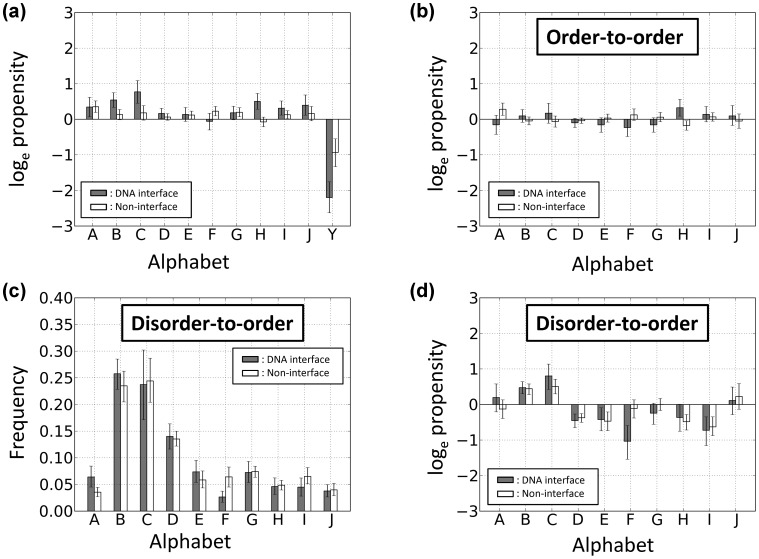
Propensities of the structural alphabets with various conditions. (a) Propensities of the structural alphabets that undergo a conformational change upon DNA binding. (b) Propensities of the structural alphabets that undergo an order-to-order conformational change upon DNA binding. (c) Frequencies of the structural alphabets that undergo a disorder-to-order conformational change (Y to one of A-J) upon DNA binding. (d) Propensities of the structural alphabets that undergo a disorder-to-order conformational change (Y to one of A-J) upon DNA binding. In (a) through (d), fragments located within DNA interfaces and those within non-interfaces are shown by filled and open bar, respectively. A positive value of the propensities indicates that frequency that alphabet is observed in DNA-bound forms is higher than that in free forms. Error bar indicates the 85% bootstrap confidence interval.

Next, the alphabets that specifically increased upon DNA binding were evaluated to determine if they were different between the DNA interfaces and non-interfaces. To this end, the alphabet propensities 
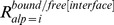
 for the DNA interfaces and 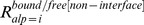 for the non-interfaces were calculated and are shown in Fig. 6(a). Here, a positive value indicates that frequency that alphabet is observed in DNA-bound forms is higher than that in free forms.

For the fragments that undergo a conformational change and are located in DNA interfaces (Fig. 6(a)), the helix-like conformation C increased most significantly (log_e_ propensity = 0.8 indicates exp(0.8) or 2.2 times more frequent in DNA interfaces than in non-interfaces). Helix-like conformations J (0.4) and I (0.3), extended conformation B (0.5), and loop-like conformations H (0.5) and A (0.3) also increased upon DNA binding. The relative frequency of Y was significantly reduced upon DNA binding (−2.2) because disorder-to-order conformational changes in the DNA interfaces often occurred upon DNA binding. In particular, the conformations B, C, and H significantly increased compared with those in the non-interfaces. In contrast to the DNA interfaces, in the non-interfaces, loop-like conformations, the values of A (log_e_ propensity = 0.4), F, and G (0.2) were positive, and these conformations were induced upon DNA binding. Helix-like conformation I and extended conformation E (0.1) also slightly increased in the DNA-bound forms, whereas Y (−0.9) was disfavored. These results indicate that disordered fragments, even in non-interfaces, tend to be ordered when they bind to DNA.

Next, the reasons why the three above-mentioned conformations increased in the DNA interfaces were considered. Initially, it was recognized that conformational changes from Y to A-J significantly increased in the DNA interfaces compared with the non-interfaces. Therefore, it was expected that the distribution of the alphabets in the DNA interfaces would be more noticeably affected by disorder-to-order conformational changes. Figure 6(b) shows the propensities of the structural alphabets that undergo an order-to-order (that is, an A-J conformation to an A-J conformation) change upon DNA binding. Regardless of whether they were in a DNA interface, the values for all alphabets were nearly zero, indicating that there is no alphabet preference except for H. H increased in the DNA interfaces, but not in the non-interfaces in these order-to-order conformational changes. Thus, the protein–DNA complex structures with H were examined and the loop-like conformation (H conformation) was identified that stabilizes the protein–DNA interactions in various ways. However, owing to the limited number of data, no common features could be identified that explain why H increases upon DNA binding.

Next, for the fragments that underwent a disorder-to-order conformational change, the frequencies of the alphabets in the DNA interfaces and non-interfaces (Fig. 6(c)) and the alphabet propensities (Fig. 6(d)) were calculated. Neither the frequencies nor the propensities of the alphabets were significantly different between the DNA interfaces and the non-interfaces. This result indicates that the structures induced from disorder-to-order conformational changes in DNA interfaces are similar to those in non-interfaces. These results suggest that changes to B or C from Y in DNA interfaces occur more frequently. Consequently, B and C are considered to be the top two preferred alphabets in disorder-order conformational changes. The reasons why the B and C conformations are favored remains a subject for future investigation.

### Conclusion

In this study, conformational changes in 4-residue fragments between DNA-free and DNA-bound forms were analyzed using structural alphabets, which enabled the precise description of the variety of local protein conformations. The results revealed the importance of the intrinsic conformational flexibility upon DNA binding: (1) intrinsic conformational variations in DNA interfaces are more frequent than those in non-interfaces and (2) conformationally changed fragments in DNA interfaces favor the disorder-promoting amino acids. In addition, it was found that three specific alphabets appeared in the DNA interfaces; however, the roles of the conformations in DNA binding are various. These findings may contribute to the more accurate prediction of the DNA binding sites of proteins and the potential conformational changes in the complex form.

## Supporting Information

Table S1 The coordinates of the fragment library reported by Kolodny *et al*.
(DOCX)

Table S2 PDB IDs and chains in DB_free_, DB_bound_ and DB_free>2_.
(XLSX)

Text S1 Supporting methods.
(DOCX)
