# Supplementary material for: Local Conformational Changes in the DNA Interfaces of Proteins
Source: PLoS One. 2013 Feb 13;8(2):e56080. doi: 10.1371/journal.pone.0056080 (PMC3571985; doi:10.1371/journal.pone.0056080)
Supplement: Table S1 — The coordinates of the fragment library reported by Kolodny et al. (DOCX) [file pone.0056080.s001.docx]

| **Alphabet** | **Cα position** | **Coordinate** | | |
| --- | --- | --- | --- | --- |
|  |  | **x** | **y** | **z** |
| **A** | 1 | 27.959 | 21.274 | 26.228 |
|  | 2 | 29.513 | 24.404 | 24.903 |
|  | 3 | 26.472 | 26.718 | 25.049 |
|  | 4 | 23.188 | 26.848 | 26.971 |
| **B** | 1 | 29.291 | 20.922 | 26.312 |
|  | 2 | 27.84 | 23.915 | 24.455 |
|  | 3 | 26.907 | 27.138 | 26.287 |
|  | 4 | 23.094 | 27.269 | 26.097 |
| **C** | 1 | 27.149 | 21.745 | 26.186 |
|  | 2 | 29.337 | 24.802 | 25.57 |
|  | 3 | 26.385 | 26.775 | 24.169 |
|  | 4 | 24.261 | 25.921 | 27.225 |
| **D** | 1 | 29.968 | 21.012 | 25.934 |
|  | 2 | 27.653 | 23.677 | 24.592 |
|  | 3 | 26.387 | 26.475 | 26.841 |
|  | 4 | 23.124 | 28.079 | 25.784 |
| **E** | 1 | 29.649 | 20.672 | 25.448 |
|  | 2 | 28.783 | 24.347 | 25.381 |
|  | 3 | 25.219 | 25.426 | 26.073 |
|  | 4 | 23.481 | 28.798 | 26.25 |
| **F** | 1 | 27.983 | 21.175 | 25.812 |
|  | 2 | 29.118 | 24.821 | 25.913 |
|  | 3 | 26.082 | 26.327 | 24.179 |
|  | 4 | 23.948 | 26.921 | 27.247 |
| **G** | 1 | 28.818 | 20.953 | 25.413 |
|  | 2 | 28.975 | 24.697 | 26.05 |
|  | 3 | 25.563 | 25.597 | 24.701 |
|  | 4 | 23.776 | 27.996 | 26.988 |
| **H** | 1 | 28.316 | 21.037 | 25.754 |
|  | 2 | 29.388 | 24.699 | 25.617 |
|  | 3 | 25.943 | 26.001 | 24.669 |
|  | 4 | 23.485 | 27.507 | 27.11 |
| **I** | 1 | 27.329 | 21.619 | 26.321 |
|  | 2 | 29.402 | 24.601 | 25.202 |
|  | 3 | 26.515 | 26.987 | 24.525 |
|  | 4 | 23.886 | 26.037 | 27.103 |
| **J** | 1 | 27.489 | 21.464 | 26.586 |
|  | 2 | 28.953 | 24.468 | 24.838 |
|  | 3 | 26.736 | 27.523 | 24.96 |
|  | 4 | 23.955 | 25.789 | 26.768 |

* The coordinate of the library is reported in Kolodny, R., Koehl, P., Guibas, L. and Levitt, M. (2002) Small libraries of protein fragments model native protein structures accurately. *J. Mol. Biol.* **323**, 297-307.
