# Supplementary material for: Local Conformational Changes in the DNA Interfaces of Proteins
Source: PLoS One. 2013 Feb 13;8(2):e56080. doi: 10.1371/journal.pone.0056080 (PMC3571985; doi:10.1371/journal.pone.0056080)
Supplement: Text S1 — Supporting methods. (DOCX) [file pone.0056080.s003.docx]

**The details for propensities calculations**

***Alphabet propensity for the conformationally changed fragments***

To investigate the characteristics of the alphabets induced by conformational changes upon DNA binding, the alphabet propensity was calculated. The probability of alphabet *i* being assigned to fragment *l* from a DNA-bound form ($W_{chg,i}^{l}$) was calculated as

$$W_{chg,i}^{l}=\frac{N_{i}^{l}}{\sum_{i=1}^{11} N_{i}^{l}}\overline{P_{i}^{l}}$$

where $P_{i}^{l}$ is the probability of fragment *l* having alphabet *i* in the DNA-free form and was calculated as

$P_{i}^{l}=\frac{n_{i}^{l}}{\sum_{i=1}^{11} n_{i}^{l}}$, $\overline{P_{i}^{l}}=1-P_{i}^{l}$.

The relative frequencies of alphabet *i* occurring in the conformationally changed fragments of the DNA interfaces from DB_free_ ($f_{alp=i}^{interface}$) and DB_bound_ ($F_{alp=i}^{interface}$) were expressed as

$f_{alp=i}^{interface}=\sum_{l\in interface} w_{chg,i}^{l}/\sum_{l\in interface} \sum_{i=1}^{11} w_{chg,i}^{l}$ and $F_{alp=i}^{interface}=\sum_{l\in interface} W_{chg,i}^{l}/\sum_{l\in interface} \sum_{i=1}^{11} W_{chg,i}^{l}$

The corresponding frequencies of the non-interfaces from DB_free_ ($f_{alp=i}^{non-interface}$) and DB_bound_ ($F_{alp=i}^{non-interface}$) were calculated as

$$f_{alp=i}^{non-interface}=\sum_{l\in non-interface} w_{chg,i}^{l}/\sum_{l\in non-interface} \sum_{i=1}^{11} w_{chg,i}^{l}$$

and

$$F_{alp=i}^{non-interface}=\sum_{l\in non-interface} W_{chg,i}^{l}/\sum_{l\in non-interface} \sum_{i=1}^{11} W_{chg,i}^{l}$$

Finally, the alphabet propensities in the DNA interfaces ($R_{alp=i}^{bound/free[interface]}$) and non-interfaces ($R_{alp=i}^{bound/free[non-interface]}$) were given by

$R_{alp=i}^{bound/free[interface]}=\frac{f_{alp=i}^{interface}}{F_{alp=i}^{interface}}$ and $R_{alp=i}^{bound/free[non-interface]}=\frac{f_{alp=i}^{non-interface}}{F_{alp=i}^{non-interface}}$

***Alphabet propensities for disorder-to-order conformationally changed fragments and order-to-order conformationally changed fragments***

To analyze the effects of order-to-order and disorder-to-order conformational changes, the alphabet propensities in these two types of conformational changes were calculated. First, the probabilities that fragment *l* has a different alphabet *i* in DNA-bound forms and DNA-free forms for the sets of order-to-order and disorder-to-order changes were calculated using the following equations:

$W_{order-order,i}^{l}=\frac{N_{i}^{l}}{\sum_{i} N_{i}^{l}}\overline{P_{order-order,i}^{l}}$ and $W_{disorder-order,i}^{l}=\frac{N_{i}^{l}}{\sum_{i} N_{i}^{l}}\overline{P_{disorder-order,i}^{l}}$,

where $P_{order-order,i}^{l}$ and $P_{disorder-order,i}^{l}$ are the probability of fragment *l* having alphabet *i* in the DNA-free form in order-to-order and disorder-to-order changes, respectively, and were calculated as

$P_{order-order,i}^{l}=\frac{n_{i}^{l}+n_{Y}^{l}}{\sum_{i}^{11} n_{i}^{l}}$, $\overline{P_{order-order,i}^{l}}=1-P_{order-order,i}^{l}$

and

$P_{disorder-order,i}^{l}=\frac{n_{Y}^{l}}{\sum_{i}^{11} n_{i}^{l}}$, $\overline{P_{disorder-order,i}^{l}}=1-P_{disorder-order,i}^{l}$

Next, the probabilities of the conformational changes of fragment *l* from the DNA-free forms in order-to-order changed were calculated using the following equation:

$$w_{order-order,i}^{l}=\frac{n_{i}^{l}}{\sum_{i} n_{i}^{l}}\overline{p_{order-order,i}^{l}}$$

where $p_{order-order,i}^{l}$ is given by

$p_{order-order,i}^{l}=\frac{N_{i}^{l}+N_{Y}^{l}}{\sum_{i} N_{i}^{l}}$, $\overline{p_{order-order,i}^{l}}=1-p_{order-order,i}^{l}$

Using these probabilities, the relative frequencies of the order-order conformational changes and disorder-order conformational changes and the alphabet propensities for the order-to-order conformational changes were calculated using the same procedures as described in the above section.

For the order-to-order conformational changes, the relative frequencies of alphabet *i* for the conformationally changed fragments from DB_free_ ($f_{order-order,alp=i}^{interface}$ for DNA interfaces and $f_{order-order,alp=i,}^{non-interface}$ for non-interfaces) and DB_bound_ ($F_{order-order,alp=i}^{interface}$for DNA interfaces and $F_{order-order,alp=i}^{non-interface}$ for non-interfaces) were given by

$f_{order-order,alp=i}^{interface}=\sum_{l\in interface} w_{order-order,i}^{l}/\sum_{l\in interface} \sum_{i=1}^{10} w_{order-order,i}^{l}$,$f_{order-order,alp=i}^{non-interface}=\sum_{l\in non-interface} w_{order-order,i}^{l}/\sum_{l\in non-interface} \sum_{i=1}^{10} w_{order-order,i}^{l}$ $F_{order-order,alp=i}^{interface}=\sum_{l\in interface} W_{order-order,i}^{l}/\sum_{l\in interface} \sum_{i=1}^{10} W_{order-order,i}^{l}$

and

$$F_{order-order,alp=i}^{non-interface}=\sum_{l\in non-interface} W_{order-order,i}^{l}/\sum_{l\in no-interface} \sum_{i=1}^{10} W_{order-order,i}^{l}$$

where $w_{order-order,i}^{l}$ and $w_{order-order,i}^{l}$ in the denominator were summed up for alphabets A-J.

In a similar manner, the alphabet propensities for the DNA interfaces ($R_{order-order,alp=i}^{bound/free[interface]}$) and non-interfaces ($R_{order-order,alp=i}^{bound/free[non-interface]}$) were given by

$R_{order-order,alp=i}^{bound/free[interface]}=\frac{F_{order-order,alp=i}^{interface}}{f_{order-order,alp=i}^{interface}}$ and $R_{order-order,alp=i}^{bound/free[non-interface]}=\frac{F_{order-order,alp=i}^{non-interface}}{f_{order-order,alp=i}^{non-interface}}$

For the disorder-to-order conformational changes, the relative frequencies of alphabet *i* in the conformationally changed fragments from DB_bound_ ($F_{disorder-order,alp=i}^{interface}$for DNA interfaces and $F_{disorder-order,alp=i}^{non-interface}$ for non-interfaces) were calculated as

$$F_{disorder-order,alp=i}^{interface}=\sum_{l\in interface} W_{disorder-order,i}^{l}/\sum_{l\in interface} \sum_{i=1}^{10} W_{disorder-order,i}^{l}$$

and

$$F_{disorder-order,alp=i}^{non-interface}=\sum_{l\in non-interface} W_{disorder-order,i}^{l}/\sum_{l\in no-interface} \sum_{i=1}^{10} W_{disorder-order,i}^{l}$$

The alphabet propensities of the fragments that undergo a disorder-to-order conformational change upon DNA binding were calculated for the DNA interfaces ($R_{disorder/order,alp=i}^{bound/free[interface]}$) and non-interfaces ($R_{disorder/order,alp=i}^{bound/free[non-interface]}$) as

$R_{disorder/order,alp=i}^{bound/free[interface]}=\frac{F_{disorder-order,alp=i}^{interface}}{f_{alp=i}^{interface}}$ and $R_{disorder/order,alp=i}^{bound/free[non-interface]}=\frac{F_{disorder-order,alp=i}^{non-interface}}{f_{alp=i}^{non-interface}}$

where $f_{alp=i}^{interface}$and $f_{alp=i}^{non-interface}$ were calculated using the conformationally changed fragments with alphabets A-J.
